# Is adolescent body mass index and waist circumference associated with the food environments surrounding schools and homes? A longitudinal analysis

**DOI:** 10.1186/s12889-018-5383-z

**Published:** 2018-05-02

**Authors:** Mark A. Green, Duncan Radley, Nik Lomax, Michelle A. Morris, Claire Griffiths

**Affiliations:** 1 Department of Geography & Planning, University of Liverpool, Liverpool, UK; 2 Carnegie School of Sport, Leeds Beckett University, Leeds, UK; 3 School of Geography, University of Leeds, Leeds, UK; 4 Leeds Institute of Data Analytics, University of Leeds, Leeds, UK; 5 Leeds Institute for Biomedical and Clinical Services, University of Leeds, Leeds, UK

**Keywords:** Obesity, Adolescents, Diet, Fast food, Geography, UK

## Abstract

**Background:**

There has been considerable interest in the role of access to unhealthy food options as a determinant of weight status. There is conflict across the literature as to the existence of such an association, partly due to the dominance of cross-sectional study designs and inconsistent definitions of the food environment. The aim of our study is to use longitudinal data to examine if features of the food environment are associated to measures of adolescent weight status.

**Methods:**

Data were collected from secondary schools in Leeds (UK) and included measurements at school years 7 (ages 11/12), 9 (13/14), and 11 (15/16). Outcome variables, for weight status, were standardised body mass index and standardised waist circumference. Explanatory variables included the number of fast food outlets, supermarkets and ‘other retail outlets’ located within a 1 km radius of an individual’s home or school, and estimated travel route between these locations (with a 500 m buffer). Multi-level models were fit to analyse the association (adjusted for confounders) between the explanatory and outcome variables. We also examined changes in our outcome variables between each time period.

**Results:**

We found few associations between the food environment and measures of adolescent weight status. Where significant associations were detected, they mainly demonstrated a positive association between the number of amenities and weight status (although effect sizes were small). Examining changes in weight status between time periods produced mainly non-significant or inconsistent associations.

**Conclusions:**

Our study found little consistent evidence of an association between features of the food environment and adolescent weight status. It suggests that policy efforts focusing on the food environment may have a limited effect at tackling the high prevalence of obesity if not supported by additional strategies.

## Background

While trends in childhood obesity may have recently stabilised in some countries [1, 2], high prevalence rates still represent a pressing public health issue. In the UK 19.1% of children aged between 10 and 11 years old are estimated to be obese, with a further 14.1% overweight [3]. Prevalence rates increase further into adolescence with 37.8% of boys and 36.6% of girls aged 11–15 years old overweight or obese [4]. Childhood obesity is associated with several adverse physical and mental health outcomes including poor metabolic outcomes, type 2 diabetes and low self-esteem both during childhood and later in the life course [5–9]. Children who are obese are also more likely to remain obese into adulthood [10].

Childhood obesity is a complex issue with multifactorial drivers including: psychological, activity and exercise levels, dietary, physiological, societal and environmental influences [11]. Identifying characteristics of this complex web that are modifiable is key for designing effective strategies to tackle childhood obesity. One of the potential environmental influences is the level of access to “unhealthy” foods surrounding an individual’s home or school. It has been hypothesised that where there is a greater supply of food outlets selling foods that are energy dense and nutritionally poor, adolescents may be more likely to consume such foods resulting in poorer diets and ultimately excess body weight [12]. Some policy makers have begun to react to this theory by restricting the location of new fast food outlets believing it will limit accessibility to unhealthy foods [13–16]. However, the policy focus on fast food outlets is far too narrow since it only encapsulates one aspect of the broader food environment. Policies should consider all food outlets (e.g. convenience stores), as well as national/global food systems that may promote unhealthy food options, to be truly effective.

Policy actions in the role of the food environment run contrary to the quality of evidence available demonstrating an association with childhood obesity. While some studies have reported a positive association between the density of fast food outlets and risk of childhood obesity [17–20], a comparable number have not found any association [21, 22]. Similarly, systematic reviews have demonstrated mixed findings for adults [23, 24]. The lack of consensus may be partly due to the reliance on cross-sectional data, poor study design or inconsistencies in methodological approaches. There has also been greater focus on the environment surrounding the home, with few studies examining the environment surrounding schools or the environment individuals may encounter when travelling between the two.

Using longitudinal data for Leeds, UK, our study aims to explore how the home and school food environments (including estimated travel routes in-between) are associated with changes in weight status throughout adolescence (ages 11/12 to 15/16 years).

## Methods

### Data source

Data were collected as part of the Rugby League and Athletics Development Scheme (RADS) which has been described previously [25]. In brief, all secondary schools in Leeds, UK (*n* = 40), were invited to take part in the programme which was initially aimed at children in their first year of secondary school (age 11–12 years). Seven schools (out of the 33 schools originally participating in RADS) agreed to participate in follow up measures in year 9 (aged 13–14 years) and year 11 (aged 15–16 years) with participation rates at the individual level being consistently high (> 90% at each measurement occasion in each of the 7 schools). There was little difference in the sociodemographic characteristics of participants from the original 33 participating schools (mean ‘Income Deprivation Affecting Children Index’ score 0.25 (see later section for more details), standard deviation 0.20) when compared to the seven schools with follow up data at baseline (mean score 0.26, standard deviation 0.22). Measurements were taken in September 2005 (year 7), January 2008 (year 9) and January 2010 (year 11), therefore, the time interval between measurements may vary slightly. All children providing consent from the seven schools were eligible to take part, however, only children with at least 2 measurements were included in the analysis. 336 (45%) children had measurements from all three years of data collection (i.e. ‘complete data’ allowing a direct comparison of exactly the same pupils over all 3 assessment occasions). An additional 410 (55%) children had at least two measurements (Y7 and Y9 *n* = 254 (34%); Y7 and Y11 *n* = 87 (12%); Y9 and Y11 *n* = 69(9%)) resulting in a final sample size of 746 children (i.e. mixed data). We tested for differences between these individuals to assess if it is an issue with our analyses. Ethical clearance was granted by the Ethics Committee of the Carnegie Faculty, Leeds Beckett University. Parental consent was obtained for participants aged under 16 years.

### Measures

#### Personal measures

Age, sex, home postcode, school postcode and race (defined as ‘White’ or ‘non-White’) were provided by the schools from their administrative databases.

#### Anthropometric measures

Body mass (kg), height (m) and waist circumference (WC) (cm) were objectively measured by the same person (CG; [25]). Body mass was measured using manually calibrated electronic scales (Tanita TBF-310, Tanita Corp, Tokyo, Japan), and children wore only light physical education clothing without shoes. Height was measured using a floor-standing Leicester height measure (children were not wearing shoes). Waist circumference was measured using an inelastic tape mid-way between the 10th rib and the iliac crest (children were wearing a thin shirt; we subtracted 0.5 cm from the value to account for this). More specific details of the data collection process can be viewed elsewhere [25]. Body mass Index (BMI) measurements were calculated and standardised (BMI SDS) for age and sex using the British 1990 growth reference charts (UK90) [26]. WC measurements were standardised (WC SDS) for age and sex using the published reference based on the data from the British Standards Institute survey [27]. While they are common measures in population research since they provide a valid measure of relative body weight [28], they do not directly measure adiposity limiting the conclusions we can draw. To ensure consistency in data collection, measurements at each time point were carried out by the same person (CG) using the same equipment and procedures.

#### Exposure to food outlets

The study builds on previous research using cross sectional data from the RADs programme [22]. We utilise the same methods to determine the exposure to food outlets to investigate if longitudinal changes in BMI and WC was associated with the food outlet availability in the home, school and home-school commute environments. Data on food outlet locations were sourced from Leeds City Council (LCC) covering all licensed premises in the study area during the time of data collection in 2005 and mapped by postcode centroid (postcode centroids are point estimates of polygons that contain a collection of street-level addresses (mean 15 addresses)). Longitudinal food outlet data were not available for the period. Food outlets were categorised into three groups, supermarkets, takeaways and other retail (e.g. newsagents, bakeries, petrol stations) according to the definitions used by LCC (outlets are categorised using a national classification based on their commercial activity; for example, takeaways were defined as ‘A3’ properties i.e. where hot food is sold for consumption on or off site). Home and school exposures were defined as circular buffers with a 1 km Euclidean (straight line) radius, centred on their postcode location and the number of food outlets falling within the buffer were identified. A 1 km buffer was selected based on previous research [28], since it represents roughly a 20 min walk which is a reasonable measure of accessibility. Travel between home and school was estimated by calculating the shortest distance on the road network using the R package ‘ggmap’ (i.e. using the Google Maps Network features). Consistent with the previous literature [23], in order to capture varying routes a 500 m buffer was placed around this route and the number of food outlets falling within this buffer were identified. A smaller buffer for the travel route was used to capture the uncertainty in the travel route, as well as to account for potential detours pupils may take to access food. Each exposure variable was measured as a continuous variable. We define the school, home and commuting food environments used in subsequent analyses as the count of each outlet type surrounding the geographical vicinity.

#### Sociodemographic measures

Social disadvantage was assessed using the Income Deprivation Affecting Children Index (IDACI), developed as part of the English Indices of Deprivation (2007) which represent a set of relative measures of neighbourhood deprivation. IDACI estimates the proportion of children under the age of 16 years in a neighbourhood that live in low income households. Since both adolescent obesity and density of fast food outlets are higher in poorer neighbourhoods [20, 23, 29], this is an important confounder to account for and as such we included both the IDACI score of the home and school environments. IDACI was measured at the Lower Super Output Area (LSOA) for each individual’s home and school (determined by postcode). LSOAs are small administrative areas that contain an average of 1500 people.

### Statistical analysis

Multi-level linear regression models were fit to examine the association between the outcome measures (BMI SDS and WC SDS) and measures of the food environment within the home, school and commuting environments (adjusting for age, sex, race and deprivation). Individual observations at each time point (level-1) were nested within individuals (level-2), who were nested within schools (level-3). Centering was not performed on any variable at any level. We also compared differences in sample characteristics for individuals with two or three observations using t-tests for continuous data (age and deprivation measures) and chi-squared tests for categorical data (sex and race). All analyses are conducted using R statistical software.

## Results

Tables 1 and 2 present summary statistics of our sample. Unsurprisingly both unstandardized measures of child weight status (BMI and WC) increased between each time period representing the growth and development during adolescence (Table 1). There were diverging patterns in standardised BMI and WC, with mean BMI SDS declining throughout adolescence and WC SDS increasing (this has been reported previously; [25]). There was little difference in patterns of exposure to each feature of the food environment between the home, school and travel environments (Table 2). Fast food outlets were more commonplace in each environment, with supermarkets least common. There was less variation around schools in our measures compared to the home and travel environments.

**Table 1:** Descriptive statistics of sample characteristics

	Mean	Standard deviation
*Year 7*		
BMI (kg/m^2^)	19.09	3.40
BMI SDS	0.40	1.19
WC (cm)	66.58	9.07
WC SDS	0.82	1.23
Age	11.59	0.30
Male (proportion)	0.48	0.50
None-White (proportion)	0.23	0.42
Home deprivation Score	0.26	0.22
School deprivation score	0.22	0.03
*Year 9*		
BMI (kg/m^2^)	20.48	3.76
BMI SDS	0.33	1.19
WC (cm)	73.95	10.47
WC SDS	1.17	1.24
Age	13.94	0.31
Male (proportion)	0.48	0.50
None-White (proportion)	0.23	0.42
Home deprivation Score	0.26	0.22
School deprivation score	0.22	0.05
*Year 11*		
BMI (kg/m^2^)	21.32	3.56
BMI SDS	0.22	1.17
WC (cm)	78.61	9.08
WC SDS	1.40	1.13
Age	15.92	0.31
Male (proportion)	0.49	0.50
None-White (proportion)	0.23	0.42
Home deprivation Score	0.26	0.22
School deprivation score	0.23	0.03

NB. *BMI* body mass index, *SDS* standardised, *WC* waist circumference

**Table 2:** Descriptive statistics of the food environment

	Median count	Interquartile range
Home (1 km)		
Fast food outlets	12	5–21
Supermarkets	2	1–3
Other retail outlets	9	5–18
School (1 km)		
Fast food outlets	12	4–14
Supermarkets	3	1–3
Other retail outlets	7	6–9
Travel (500 m)		
Fast food outlets	11	7–23
Supermarkets	2	1–4
Other retail outlets	9	4–20

Table 3 compared differences in sample characteristics between individuals who had two or three observations. No difference was found for age or race, but we detect differences for sex (individuals with three observations were more likely to be male) and deprivation (individuals with only two observations were more likely to live in more deprived areas). These differences should be considered with the interpretation of our results.

**Table 3:** Differences in sample characteristics between individuals with two or three observations

	Mean values		Tests	
	2 observations	3 observations	Statistic	*p*-value
Age during Year 7	11.6	11.6	0.80	0.427
Age during Year 9	13.9	13.9	1.28	0.200
Age during Year 11	15.9	15.9	1.47	0.144
Sex (Male)	0.44	0.54	7.37	0.007
Race (Non-White)	0.24	0.21	0.90	0.343
Home deprivation Score	0.29	0.22	4.07	< 0.001
School deprivation score	0.25	0.18	5.80	< 0.001

NB. T-tests were performed for continuous data (and mean values are presented in the table), chi-squared tests for categorical (proportions are presented in the table)

Table 4 presents the results from our regression model examining the association of the food environment with BMI SDS and WC SDS (Table 2). Few associations were found where the 95% confidence intervals did not cross zero. For BMI SDS, a positive association between the number of ‘other retail outlets’ within 1 km of a school whereby an additional outlet is associated with an increase of BMI SDS of 0.038 (95% CIs 0.006–0.0052). For WC SDS we detected a positive association for the number of fast food outlets along the travel route, with an additional fast food outlet associated with an increase on 0.021 (95% CIs 0.007–0.033). We also found a negative association for ‘other retail outlets’ along the travel route (Coefficient = − 0.014, 95% CIs − 0.027 - -0.001). All other associations across both models were non-significant.

**Table 4:** Fixed effects parameters of multi-level models examining environmental predictors of standardised body mass index and waist circumference

Variable	BMI SDS		WC SDS	
	Coefficient	95% CIs	Coefficient	95% CIs
Home (1 km)				
Fast food outlets	−0.017	(−0.035, 0.002)	*−0.024*	*(−0.041, −0.006)*
Supermarkets	0.021	(−0.022, 0.090)	0.037	(− 0.013, 0.094)
Other retail outlets	0.003	(−0.014, 0.017)	0.007	(− 0.008, 0.021)
School (1 km)				
Fast food outlets	−0.020	(−0.057, 0.057)	− 0.010	(− 0.184, 0.167)
Supermarkets	− 0.086	(− 0.372, 0.084)	−0.035	(− 0.730, 0.655)
Other retail outlets	*0.038*	*(0.006, 0.052)*	0.031	(−0.046, 0.106)
Travel route (500 m)				
Fast food outlets	0.017	(−0.001, 0.027)	*0.021*	*(0.007, 0.033)*
Supermarkets	0.014	(−0.033, 0.057)	−0.017	(− 0.059, 0.025)
Other retail outlets	−0.015	(− 0.027, 0.001)	−0.014	(− 0.027, − 0.001)
Intra-Class Correlation				
Year	0.000		0.144	
School	0.054		0.203	

NB. Adjusted for age, sex, race and level of deprivation in home and school environments. Yearly observations (level 1) were nested within individuals (level 2) which were nested within schools (level 3). *CIs* Confidence Intervals, *BMI SDS* standardised body mass index, *WC SDS* standardised waist circumference, We have used italics to indicate those results where the CIs do not cross a value of 0

## Discussion

### Key results

Using longitudinal data, our findings demonstrate few associations between the food environment and measures of adolescent weight status. Where we detected associations, they mostly fell in the hypothesised direction. The presence of a fast food outlet along the estimated travel route between school and home, and the presence of ‘other retail outlets’ near schools were both positively associated with weight status. While upon initial inspection their effect sizes (i.e. coefficients) appear small and not clinically significant, they refer to the change in weight status for an additional outlet. When considered alongside an additional 10 outlets, the effect size is clinically significant. Such a finding would be appropriate for the number of fast food outlets along the travel route since the interquartile range (IQR) was 7–23 (although the coefficient was not significant for BMI SDS). However, for the number of ‘other retail outlets’ it is a less suitable observation since there does not appear to be this magnitude of variation (IQR 6–9).

### Limitations

Longitudinal data were not used for the exposure variables since they were not available. Food retail environments do not change quickly (particularly in comparison to the time period of our study), suggesting that our data are appropriate and the impact of bias on our results will be low [30]. Information on key covariates such as diet, physical activity or parental characteristics, which have all been shown to be associated with adolescent weight status, were not available. These unknown characteristics may confound the associations observed. Data collection was also unbalanced, with varying sample sizes between years which may introduce bias into our estimates.

We used buffers to measure exposure to aspects of the food environment. It is unlikely that these buffers correspond to the actual food environments individuals engage and interact with [31]. While we find little evidence of the role of geographical context in our study, the role geography plays is complex and likely to operate at different interacting scales (i.e. local vs regional food environments). It is also difficult to measure the exact environments individuals engage in without using GPS data. Our use of buffers was to provide a proxy measure to capture different routes or uses of environments, but they are only estimates. As such, we should acknowledge that the use of single point estimates to measure the ‘home’, ‘school’ and ‘commuting’ environments may introduce the locational fallacy, since we do not know the exact exposure of individuals to their food environments. We did calculate smaller buffers (500 m) for the home and school environment to test the impact of the more immediate environment, however this did not alter our findings (results not shown). Travel routes were also estimated since the data were not available limiting the observations that can be inferred from them. Since one of the positive associations found was along the travel route, we can only suggest that this is an interesting avenue for future research to explore in greater detail.

Our study focuses only on one aspect of accessibility (i.e. geographical proximity), however it is important to account for broader aspects of the food environment. Home and school characteristics themselves have direct impacts of dietary choices [32], and they may influence how individuals engage with surrounding neighbourhood features. In this vein, characterising the types of food sold by outlets, the price of food options (also linked to an individual’s food purchasing power) or their opening hours will be important in providing greater detail on the role of environmental features.

Finally, our study was purely descriptive in exploring the association between the density of features of the food environment and measures of weight status. While this is useful for initially understanding if any association(s) exist, and follows previous studies (e.g. 15,28), there is greater need in future research to explore the causal pathways and mechanisms through which the food environment may influence weight status.

### Interpretation

Our study makes a novel contribution to the literature building on previous studies by using longitudinal data across the period of secondary school, an important period in the anthropometric development in children and one which is key for their risk of obesity during adolescence and into adulthood [10]. We examined two measures of weight status and multiple spatial contexts (home, school and travel route environments) to capture the wider experience of food environments.

The majority of studies examining the role of food environments on weight status are cross-sectional and therefore less able to draw out cause and effect (even if analyses are carried out within a causal framework). Findings from cross-sectional studies are ambiguous with some reporting positive associations between fast food outlets and weight status [17–20], but a comparable number finding no association (19–21). The lack of consistency across the literature may be a symptom of residual confounding across studies, suggesting the importance of correct study design when using observational data [33]. A null association may simply be because there is too much ‘noise’ in the data to be able to observe the true effect. It is important for future research to develop stronger causal models that can be evaluated will help to lessen the impact of residual confounding.

To our own knowledge, our study is the first longitudinal analysis using UK data. Sturm and Datar [21] investigated the association between food outlet density and changes in BMI over 1 and 3 years among elementary school children in the USA, finding null associations [21]. Although interestingly they did report differential gains in BMI according to geographical variation in fruit and vegetable prices, possibly suggesting that additional measures beyond simple density metrics are required to expose geographical associations. Adolescents develop at different stages, so taking a longitudinal approach is more appropriate than a snap shot of cross sectional data. In addition to changes in physical development, adolescent behaviours are likely to change during this period as they transition toward independence. The longitudinal data will go some way to encapsulate this. That being said, our results using longitudinal data do match results from the baseline cross-sectional analysis reported previously [22].

Our findings have important policy implications. The inconsistency of evidence from our study and throughout the literature between the density of fast food outlets and weight status suggests that efforts to restrict the locations of new fast food outlets may be of limited value as a standalone intervention to tackle childhood obesity [13–16]. We do not contest that strategies to improve the food environment surrounding homes and school are logical. There have been long term shifts of increasing access to unhealthy foods corresponding to increased prevalence of obesity [30]. Rather policies need to be move beyond simply restricting their numbers alone. Table 2 demonstrated that while in our study the median number of fast food outlets surrounding homes, schools and travel route was similar (12, 12 and 11 respectively), the interquartile ranges suggests that there was considerable variability. It could be hypothesised that restricting the location of new fast food outlets in areas with few fast food outlets may have a larger effect (assuming that a community has enough food sources). However, the majority of areas could already be at a level of saturation whereby restricting further outlets will likely have little impact.

If Local Authorities are unable to make significant changes to the spatial availability of unhealthy foods, then they need to consider alternative strategies dealing with the existing environments. Licensing may offer one approach that could be effective at addressing the whole food environment, such as reducing opening hours, encouraging firms to use less cooking oil or introducing price subsidies for healthy food options [34–36]. Similar restrictive licencing policies for alcohol sale, implemented at the local authority level [37], have been successful in reducing purchases and consumption. In terms of national policy, minimum pricing of alcohol has been estimated to reduce alcohol consumption and subsequent health-related harms [38]; similar policy interventions directed towards fast food outlets may help reduce the purchase and consumption of unhealthy food. Developing local food systems which promote healthier choices, as well as dealing with wider issues such as food insecurity or the social determinants of poor dietary choices, may be more appropriate policy strategies than simply restricting the location or density of types of outlets. Examining the contribution of these policy scenarios for tackling obesity represent useful avenues for future research.

## Conclusions

Our study addresses the dearth of longitudinal evidence on the association between the food environment and adolescent weight status. We find few associations between each component of the food environment (across multiple spatial contexts of exposure) and two measures of adolescent weight status. The results suggest caution should be placed on policy interventions aimed at tackling childhood obesity through environmental features which may have limited effect when implemented in isolation from other strategies.

## Abbreviations

BMI: Body Mass Index
CI: Confidence Interval
IDACI: Income Deprivation Affecting Children Index
IQR: Interquartile Range
LCC: Leeds City Council
LSOA: Lower Super Output Area
RADS: Rugby League and Athletics Development Scheme
SDS: Standardised
WC: Waist Circumference

## References

[1] Rokholm B, Baker JL, Sørensen TIA (2010). The levelling off of the obesity epidemic since the year 1999 – a review of evidence and perspectives. Obes Rev.

[2] Wabitsch M, Moss A, Kromeyer-Hauschild K (2014). Unexpected plateauing of childhood obesity rates in developed countries. BMC Med.

[3] HSCIC. National Child Measurement Programme: England, 2014/15 school year. 2015. Available from: http://content.digital.nhs.uk/catalogue/PUB19109/nati-chil-meas-prog-eng-2014-2015-rep.pdf. Accessed 4 Apr 2018.

[4] van Jaarsveld CHM, Gulliford MC (2015). Childhood obesity trends from primary care electronic health records in England between 1994 and 2013: population-based cohort study. Arch Child Dis.

[5] Haines L, Chong Wan K, Lynn R, Barrett TG, Shield JPH (2007). Rising incidence of type 2 diabetes in children in the U.K. Diabetes Care.

[6] Cote AT, Harris KC, Panagiotopoulos C, Sandor GGS, Devlin AM (2013). Childhood obesity and cardiovascular dysfunction. J Am Coll Cardiol.

[7] Griffiths LJ, Parsons TJ, Hill AJ (2010). Self-esteem and quality of life in obese children and adolescents: a systematic review. Int J Pediatr Obes.

[8] Park MH, Falconer C, Viner RM, Kinra S (2012). The impact of childhood obesity on morbidity and mortality in adulthood: a systematic review. Obes Rev.

[9] Reilly JJ, Kelly J (2011). Long-term impact of overweight and obesity in childhood and adolescence on morbidity and premature mortality in adulthood: systematic review. Int J Obes.

[10] Monasta L, Batty GD, Cattaneo A, Lutje V, Ronfani L, Van Lenthe FJ (2010). Early-life determinants of overweight and obesity: a review of systematic reviews. Obes Rev.

[11] Government Office for Science. Tackling Obesities: Future Choices (2nd Edition) [Internet]. 2007. Available from: https://www.gov.uk/government/uploads/system/uploads/attachment_data/file/287937/07-1184x-tackling-obesities-future-choices-report.pdf. Accessed 4 Apr 2018.

[12] Bowman SA, Gortmaker SL, Ebbeling CB, Pereira MA, Ludwig DS (2004). Effects of fast-food consumption on energy intake and diet quality among children in a National Household Survey. Pediatrics.

[13] Cavill N, Rutter H. Obesity and the environment: regulating the growth of fast food outlets. London; 2014. Available from: https://www.gov.uk/government/uploads/system/uploads/attachment_data/file/296248/Obesity_and_environment_March2014.pdf. Accessed 4 Apr 2018.

[14] St. Helens Council. Supplementary planning document hot food takeaways. 2011. Available from: https://www.sthelens.gov.uk/media/3181/hot-food-takeaway.pdf. Accessed 4 Apr 2018.

[15] Dr Foster Intelligence and Land Use Consultants. Tackling the takeaways: A new policy to address fast-food outlets in Tower Hamlets. 2011. Available from: http://www.towerhamlets.gov.uk/Documents/Planning-and-building-control/Strategic-Planning/Local-Plan/Evidence-base/A5-Takeways.pdf. Accessed 4 Apr 2018.

[16] Office for the Mayor of London. Takeaways toolkit: Tools, interventions and case studies to help local authorities develop a response to the health impacts of fast food takeaways. 2012. Available from: https://www.london.gov.uk/sites/default/files/takeawaystoolkit.pdf. Accessed 4 Apr 2018.

[17] Davis B, Carpenter C (2009). Proximity of fast-food restaurants to schools and adolescent obesity. Am J Public Health.

[18] Fraser LK, Edwards KL (2010). The association between the geography of fast food outlets and childhood obesity rates in Leeds, UK. Health Place.

[19] Jennings A, Welch A, Jones AP, Harrison F, Bentham G, Van SEMF (2011). Local food outlets, weight status, and dietary intake. Am J Prev Med.

[20] Cetateanu A, Jones A (2014). Understanding the relationship between food environments, deprivation and childhood overweight and obesity: evidence from a cross sectional England-wide study. Health Place.

[21] Sturm R, Datar A (2005). Body mass index in elementary school children, metropolitan area food prices and food outlet density. Public Health.

[22] An R, School SR (2012). Residential neighborhood food environment and dietary intake among California children and adolescents. Am J Prev Med.

[23] Griffiths C, Frearson A, Taylor A, Radley D, Cooke C (2014). A cross sectional study investigating the association between exposure to food outlets and childhood obesity in Leeds, UK. Int J Behav Nutr Phys Act.

[24] Fraser LK, Edwards KL, Cade J, Clarke GP (2010). The geography of fast food outlets: a review. Int J Environ Res Public Heal.

[25] Cobb LK, Appel LJ, Franco M, Jones-Smith JC, Nur A, Anderson CAM (2015). The relationship of the local food environment with obesity: a systematic review of methods, study quality, and results. Obesity.

[26] Griffiths C, Gately P, Marchant PR, Cooke CB (2013). A five year longitudinal study investigating the prevalence of childhood obesity : comparison of BMI and waist circumference. Public Health.

[27] Cole J, Freeman JV (1995). Body mass index reference curves for the UK, 1990. Arch Dis Child.

[28] McCarthy H, Jarrett K, Crawley HF (2001). The development of waist circumference percentiles in British. Eur J Clin Nutr.

[29] Romero-Corral A, Somers V, Sieraa-Johnson J, Thomas R, Bailey K, Collazo-Clavell M (2010). Accuracy of body mass index to diagnose obesity in the US adult population. Int J Obes.

[30] Day PL, Pearce J (2011). Obesity-promoting food environments and the spatial clustering of food outlets around schools. Am J Prev Med.

[31] Maguire ER, Burgoine T, Monsivais P (2015). Health & Place Area deprivation and the food environment over time: A repeated cross-sectional study on takeaway outlet density and supermarket presence in Norfolk, UK, 1990–2008. Health Place.

[32] Dunaway LF, Carton T, Ma P, Mundorf AR, Keel K, Theall KP (2017). Beyond food access: the impact of parent-, home-, and neighborhood-level factors on Children’s diets. Int J Environ Res Public Heal..

[33] Rummo PE, Guilkey DK, Ng SW, Meyer KA, Popkin BM, Reis JP (2017). Does unmeasured confounding influence associations between the retail food environment and body mass index over time ? The coronary artery risk development in young adults ( CARDIA ) study. Int J Epidemiol.

[34] An R (2013). Effectiveness of subsidies in promoting healthy food purchases and consumption: a review of field experiments. Public Health Nutr.

[35] Mcgill R, Anwar E, Orton L, Bromley H, Lloyd-williams F, Flaherty MO (2015). Are interventions to promote healthy eating equally effective for all? Systematic review of socioeconomic inequalities in impact. BMC Public Health.

[36] Brimblecombe J, Ferguson M, Chatfi MD, Liberato SC, Gunther A, Ball K (2017). Effect of a price discount and consumer education strategy on food and beverage purchases in remote indigenous Australia: a stepped-wedge randomised controlled trial. Lancet Public Heal.

[37] De Vocht F, Heron J, Campbell R, Egan M, Mooney JD, Angus C (2017). Testing the impact of local alcohol licencing policies on reported crime rates in England. J Epidemiol Community Heal.

[38] Purshouse RC, Meier PS, Brennan A, Taylor KB, Rafi R (2010). Estimated eff ect of alcohol pricing policies on health and health economic outcomes in England : an epidemiological model. Lancet.

